# Understanding physical (in-) activity, overweight, and obesity in childhood: Effects of congruence between physical self-concept and motor competence

**DOI:** 10.1038/s41598-018-24139-y

**Published:** 2018-04-12

**Authors:** T. Utesch, D. Dreiskämper, R. Naul, K. Geukes

**Affiliations:** 1 University of Münster, Münster, Germany; 2 Willibald Gebhardt Institute, Münster, Germany

## Abstract

Both the physical self-concept and actual motor competence are important for healthy future physical activity levels and consequently decrease overweight and obesity in childhood. However, children scoring high on motor competence do not necessarily report high levels of physical self-concept and vice versa, resulting in respective (in-) accuracy also referred to as (non-) veridicality. This study examines whether children’s accuracy of physical self-concept is a meaningful predictive factor for their future physical activity. Motor competence, physical self-concept and physical activity were assessed in 3^rd^ grade and one year later in 4^th^ grade. Children’s weight status was categorized based on WHO recommendations. Polynomial regression with Response surface analyses were conducted with a quasi-DIF approach examining moderating weight status effects. Analyses revealed that children with higher motor competence levels and higher self-perceptions show greater physical activity. Importantly, children who perceive their motor competence more accurately (compared to less) show more future physical activity. This effect is strong for underweight and overweight/obese children, but weak for normal weight children. This study indicates that an accurate self-perception of motor competence fosters future physical activity beyond single main effects, respectively. Hence, the promotion of actual motor competence should be linked with the respective development of accurate self-knowledge.

## Introduction

Physical inactivity is a worldwide epidemic^1^, with one-third of the adult world population not meeting the public health guidelines for recommended levels of physical activity^2^. This tendency of growing physical inactivity is already apparent in childhood, as about 81% of the 11- to 17-year-old children and youth fail to meet their recommended physical activity levels^3^. Physical inactivity is related to increased risks for the development of chronic, non-communicable diseases such as coronary heart disease, diabetes mellitus, and cancer^4^. Particularly, the causal relationship between physical inactivity and overweight and obesity has been well established in the literature^5,6^. Already about 41 million preschool children are currently being overweight or obese^7,8^. Therefore, key priorities of the global public health policy are to counteract the early onset of obesity and to promote an active lifestyle already in childhood because physically active children have fewer subsequent weight and according health problems^9^. Hence, research has investigated various risk factors such as lack of sleep or the body mass index of parents^10^, exercise behavior with a hegemonic focus on medical approaches (e.g, caloric expenditure), dose response relations^11^, and exercise motivation or quality^12^.

From a sport and exercise sciences perspective, one key factor to promote physical activity is motor competence. In 2008, Stodden and colleagues were the first to propose a comprehensive conceptual model from a developmental perspective introducing motor competence levels as a fundamental health determinant^13^. Motor competence is hereby referred to as the ability of executing a wide variety of gross and fine motor skills^14^. It is seen as one of the principal competencies that promotes future physical activity engagement across childhood (and beyond)^15^. For instance, Barnett *et al*.^16^ showed how childhood motor competence, in particular ball skills, predict long-term sustainable physical activity and weight status six years later^17–20^. In contrast, being physically inactive in childhood is associated with difficulties in developing appropriate motor competence levels^21^, which can lead to a negative spiral of physical activity engagement^13^.

From a psychological perspective, an essential factor to promote physical activity is a positive physical self-concept (also sometimes referred to as perceived motor competence^22^). Across domains, the self-concept is one of the most powerful predictors, for instance, in health and educational psychology^23^. In sport and exercise psychology, the physical self-concept has been identified as a psychological component through which motor skill level in childhood reverberates into physically active and healthy lifestlyles later in adolescence^24^, higher self-esteem levels^25^, and physical education motivation^26^. In sum, studies have shown moderately positive (also partly prospective) associations between the physical self-concept and physical activity, which increases with age^24^. However, in their systematic review and meta-analysis, Babic *et al*.^24^ state that the role of the physical self-concept is unclear, with suggestions ranging from being a moderator or mediator variable to being an antecedent or consequence.

Hence, a large body of research regarding the promotion of physical activity and physical health in childhood focusses on two major components both assumed to have positive main effects on future physical activity: actual motor competence and the physical self-concept (or perceived motor competence). However, researchers have often investigated these main effects in isolation, thereby ignoring potential effects that result from a specific interplay of motor competence and physical self-concept^15,24^. Only recently, research has began to examine motor competence, physical self-concept, and physical activity together. For instance, Robinson *et al*.^27^ summarize that self-perceptions mediate the relationship between motor competence and physical activity, while, from a developmental (longitudinal) perspective, reciprocal effects between the physical self-concept and physical activity were found, as well as between motor competence or physical self-concept and physical activity, respectively^28^.

Competencies and related self-perceptions can be discordant, especially in childhood, due to, for instance, a lack of self-knowledge, diverse feedback, or the lack of ability for adequat social comparisons. A child who scores high on self-perception does not necessarily show high motor performance and a child who shows high motor performance scores does not necessarily score high on self-perception. Thus, self-perceiving one’s own actual competence level naturally goes along with perception errors. These perception errors result in a continuous distribution that describes individual differences in children who underestimate their motor competence levels (lower pole), who perceive themselves rather realistically or accurately (intermediate range), or who overestimate their own competence levels (upper pole). This is also referred to as the veridicality of physical self-concept, which can be described as a specific fit-pattern between actual motor competence and physical self-concept. In the literature, it is assumed that overestimation is functional to promote achievement behavior^29,30^, sport participation, physical activity^31^, and other health outcomes. In school, a slightly positive academic self-view can predict, for instance, maths achievement^32^ or, similarly, physical self-esteem^33^. However, it has also been shown that exceeding self-enhancement can lead to reduced social acceptance^34^, while a diminished self-view can result in lowered motivation, lowered achievement, and inadequate task choices^30,35^. Although the definition and operationalization of fit pattern differs across studies, the importance of considering perception accuracy as a predictor for health is supported theoretically and empirically. Hence, fit patterns of motor competence and physical self-concept might be a meaningful predictive factor for child physical activity behavior, which in turn can help to prevent weight gain^9^.

Considering these associations, the purpose of this study is to simultaneously investigate the main effects *and* the specific interplay of actual and self-perceived motor competence on future physical activity in childhood. Specifically, we are interested in whether accurate self-views (i.e., being a good/bad athlete *and* thinking of oneself as being a good/bad athlete) promote physical activity a year later above and beyond their main effects and whether these effects are moderated by weight status.

## Results

In a first step, we examined the stability of physical activity, motor competence, and physical self-concept to test whether the assessments provided reliable estimates of individual differences over time. Physical activity in 3^rd^ and 4^th^ grade were substantially associated (*r* = 0.55, *p* < 0.001). Motor competence levels were, as found in the literature and suggested by age-related improvement^27^, moderately stable from 3^rd^ to 4^th^ grade (*r* = 0.37, *p* < 0.001). Physical self-concept levels were highly correlated between 3^rd^ and 4^th^ grade (*r* = 0.54, *p* < 0.001). These results indicate reliable estimations, leaving the opportunity of successfully predicting future physical activity.

In a second step, polynomial regressions with response surface analysis were computed. The first model examination resulted in three differently constrained models within a ΔAICc range of 3. These models are summarized in Table 1: the shifted and rotated rising ridge model (SRRR), the full polynomial model (Full), as well as the rising ridge model (RR). All three models do not differ significantly and show acceptable CFI fits (>0.9), and comparable adjusted *R*^2^s. The regression weights of these model estimates are presented in Table 2.

**Table 1:** Model Comparison for the Complete Sample, Ordered by ΔAIC.

Models	k	AICc	ΔAIC	CFI	adj. *R*^2^	χ^2^	*p*_χ2_
SRRR	6	11701.40	0	>0.99	0.055	0.10	*NA*
Full	7	11703.33	1.93	>0.99	0.054	*NA*	0.779
RR	4	11704.11	2.71	0.904	0.049	6.85	0.071
SRR	5	11705.13	3.74	0.903	0.049	5.86	0.041
Additive	4	11707.33	5.93	0.82	0.045	10.72	0.017

Note. Only models with ΔAIC < 7 are shown in this Table.

k = number of parameters; AICc = corrected Akaike Information Criterion; CFI = Comparative Fit Index; adj. *R*^2^ = adjusted variance explained by the model; χ^2^ = model test of SRRR vs. model; *p*_χ_^2^ = *p*-value of comparison between the SRRR model and the respective model.

Model abbreviations: SRRR = Shifted and rotated rising ridge model; Full = full regression model; RR = Rising ridge model; SRR = Shifted rising ridge model; additive = model with two linear main effects.

**Table 2:** Regression Coeficients b_1_ to b_5_ and Derived Model Parameters (a_1_ to a_4_) for the Shifted and Rotated Rising Ridge (SRRR), the Full Polynomial (Full) and the Rising Ridge (RR) Models.

Model	Estimate	robust SE	95% CI (lower)	95% CI (upper)	*p*
**SRRR**					
b_1_	0.066	0.015	0.037	0.096	<0.001
b_2_	0.036	0.015	0.007	0.065	0.015
b_3_	−0.002	0.003	−0.008	0.004	0.561
b_4_	0.015	0.014	−0.011	0.042	0.253
b_5_	−0.034	0.013	−0.059	−0.010	0.006
LOC	0.102	0.018	0.066	0.139	<0.001
a_2_	−0.021	0.014	−0.049	0.007	0.149
a_3_	0.030	0.023	0.016	0.076	0.199
LOIC	−0.052	0.022	0.095	−0.008	0.020
**Full**					
b_1_	0.063	0.019	0.026	0.099	<0.001
b_2_	0.036	0.015	0.007	0.065	0.015
b_3_	−0.005	0.011	−0.026	0.016	0.662
b_4_	0.015	0.014	−0.012	0.042	0.269
b_5_	−0.034	0.013	−0.059	−0.010	0.006
LOC	0.099	0.021	0.057	1.141	<0.001
a_2_	−0.024	0.018	−0.060	0.012	0.197
a_3_	0.026	0.026	−0.025	0.078	0.314
LOIC	−0.054	0.023	−0.100	−0.008	0.021
**RR**					
b_1_	0.047	0.009	0.028	0.065	<0.001
b_2_	0.047	0.009	0.028	0.065	<0.001
b_3_	−0.014	0.005	−0.024	−0.003	0.011
b_4_	0.027	0.011	0.006	0.048	0.011
b_5_	−0.014	0.005	−0.024	−0.003	0.011
LOC	0.093	0.019	0.057	0.130	<0.001
a_2_	0.000	*NA*	*NA*	*NA*	*NA*
a_3_	0.000	*NA*	*NA*	*NA*	*NA*
LOIC	−0.054	0.021	−0.096	−0.012	0.011

*a_2_ (curvature effect on the LOC) and *a*_3_ (ridge shifted away from the LOC) are not modeled within the RR model.

**Figure 1:**
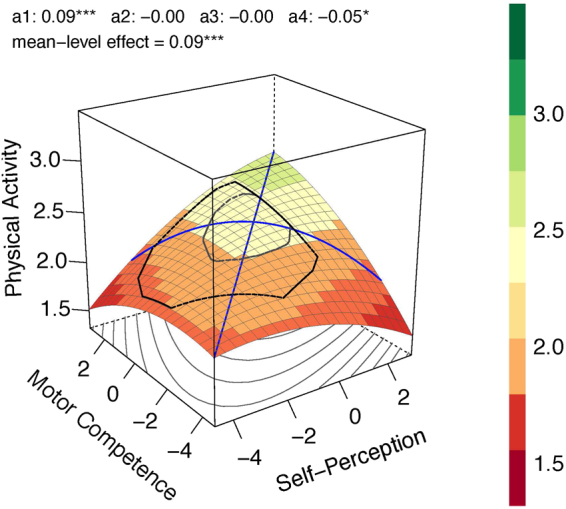
Response Surface Analysis plot for the whole sample.

All three models consistently show significant linear effects (0.047 ≤ *b*_1_ ≤ 0.066; 0.036 ≤ *b*_2_ ≤ 0.047; power > 0.94) for both predictors, a significant curvilinear effect (−0.034 ≤ *b*_5_ ≤ −0.014; power > 0.519) for motor competence, a linear effect of accuracy (0.093 ≤ *a*_1_ ≤ 0.102) as well as an incongruency effect (−0.054 ≤ *a*_4_ ≤ −0.052). The RR model additionally indicates a small, low powered (power > 0.31) curvilinear effect (*b*_3_ = −0.014) of the physical self-concept and a small, low powered (power > 0.30) interaction of both (Fig. 1).

In conclusion, higher scores in both predictors are associated with comparatively greater future physical activity levels and, additionally, more accurate self-perceptions are related to comparatively greater levels of future physical activity. The response surface plot of the RR model for the whole sample is illustrated in Fig. 1, because it is the most parsimonious model.

Quasi DIF analyses are based on the RR model (for additional Figures, data and code^36^). Analyses for the different weight groups are presented in Table 3.

**Table 3:** Regression Coeficients b_1_ to b_5_ and Derived Model Parameters (a_1_ to a_4_) the Rising Ridge (RR) Models of Underweight, Normal Weight, Overweight/Obesity.

Model	Estimate	robust SE	95% CI (lower)	95% CI (upper)	*p*
**Underweight**					
b_1_	0.083	0.027	0.030	0.136	<0.001
b_2_	0.083	0.027	0.030	0.136	0.002
b_3_	−0.065	0.016	−0.095	−0.034	0.002
b_4_	0.130	0.031	0.068	0.191	<0.001
b_5_	−0.065	0.016	−0.095	−0.034	<0.001
LOC	0.166	0.054	0.059	0.272	0.002
a_2_	*NA*	*NA*	*NA*	*NA*	*NA*
a_3_	*NA*	*NA*	*NA*	*NA*	*NA*
LOIC	−0.259	0.063	−0.382	−0.137	<0.001
**Normal weight**					
b_1_	0.035	0.012	0.012	0.058	0.003
b_2_	0.035	0.012	0.012	0.058	0.003
b_3_	−0.003	0.006	−0.014	0.007	0.533
b_4_	0.007	0.011	−0.015	0.029	0.533
b_5_	−0.003	0.006	−0.014	0.007	0.533
LOC	0.070	0.023	0.024	0.116	0.003
a_2_	*NA*	*NA*	*NA*	*NA*	*NA*
a_3_	*NA*	*NA*	*NA*	*NA*	*NA*
LOIC	−0.014	0.022	−0.057	0.030	0.533
**Overweight/Obesity**					
b_1_	0.076	0.018	0.041	0.111	<0.001
b_2_	0.076	0.018	0.041	0.111	<0.001
b_3_	−0.024	0.010	−0.043	−0.005	0.012
b_4_	0.049	0.019	0.011	0.087	0.012
b_5_	−0.024	0.010	−0.043	−0.005	0.012
LOC	0.152	0.036	0.082	0.221	<0.001
a_2_	*NA*	*NA*	*NA*	*NA*	*NA*
a_3_	*NA*	*NA*	*NA*	*NA*	*NA*
LOIC	−0.098	0.039	−0.173	−0.022	0.012

**Figure 2:**
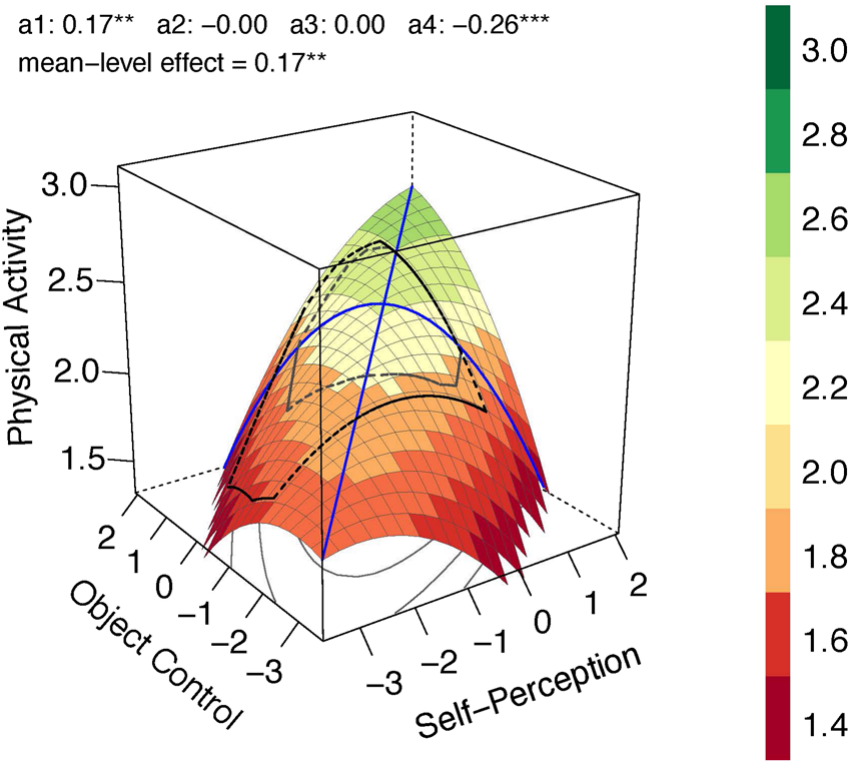
Response Surface Analysis plot for the children with underweight status.

**Figure 3:**
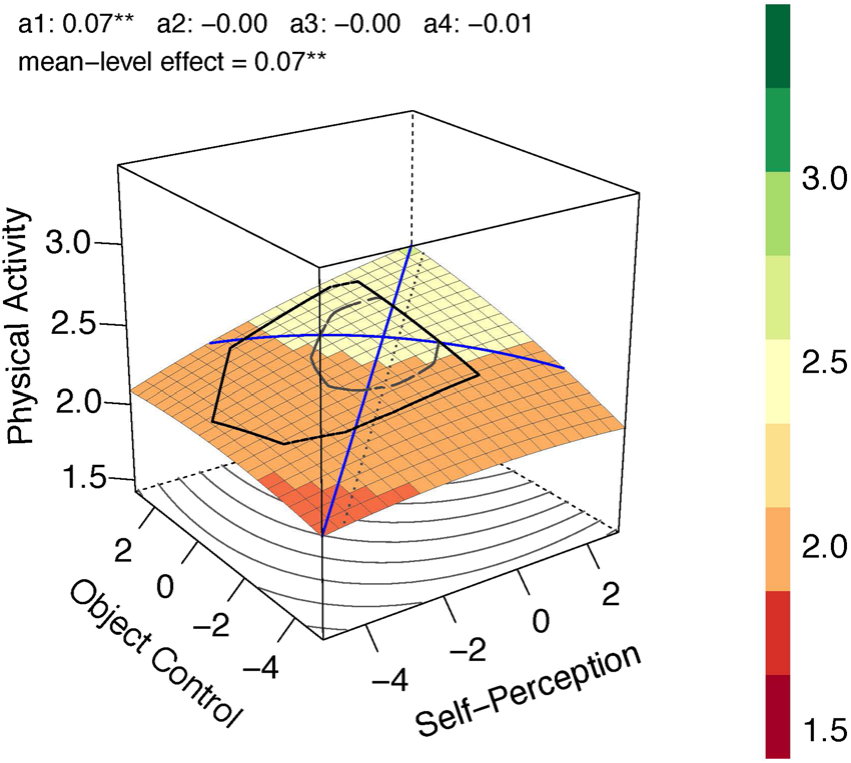
Response Surface Analysis plot for the children with normal weight status.

**Figure 4:**
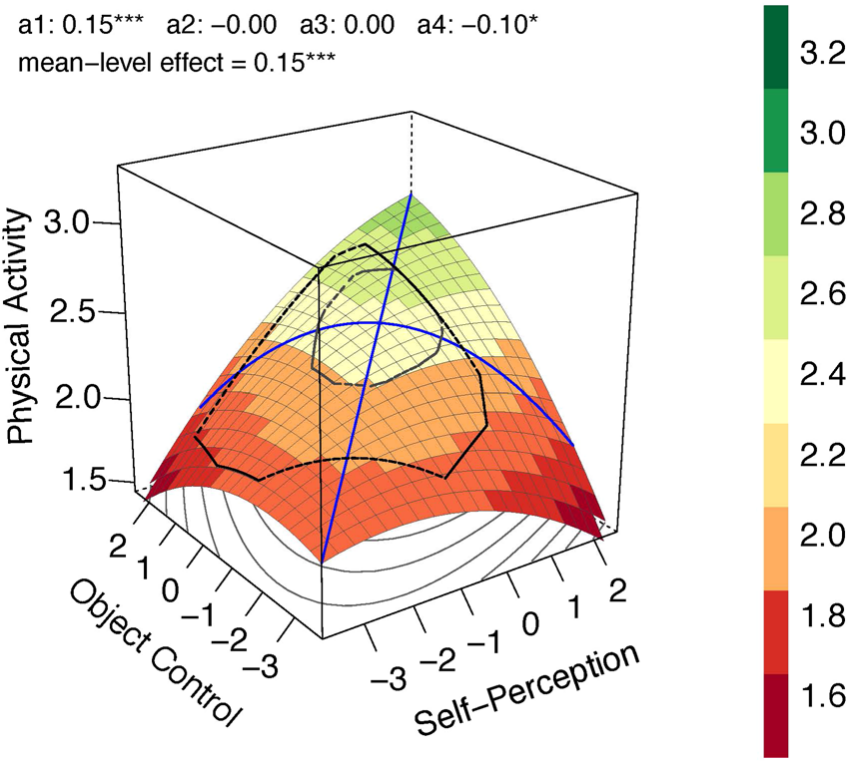
Response Surface Analysis plot for the children with overweight/obesity status.

Overweight and obese children were aggregated in these analyses to ensure enough power (for more detailed weight group-specific analyses^36^). For all three weight groups linear effects were found. However, weight seems to positively moderate the effects to a second degree as illustrated for the underweight group (Fig. 2; adj. *R*^2^ = 0.31), the normal weight group (Fig. 3; adj. *R*^2^ = 0.022) and the overweight/obesity group (Fig. 4; adj. *R*^2^ = 0.146). In the underweight (*b*_1_,*b*_2_ = 0.083; comparison to normal weight, *p* = 0.052) and overweight/obesity group (*b*_1_,*b*_2_ = 0.076; comparison to normal weight, *p* = 0.029) stronger linear effects were found compared to the normal weight group (*b*_1_,*b*_2_ = 0.035). The effects of congruence as well as of incongruence follow the same rule. The underweight (*a*_1_ = 0.166; comparison to normal weight, *p* = 0.051) and overweight/obesity groups (*a*_1_ = 0.152; comparison to normal weight, *p* = 0.028) show stronger effects compared to the normal weight group (*a*_1_ = 0.07) for congruence of motor competence and physical self-concept. Underweight (*a*_4_ = −0.259; comparison to normal weight, *p* < 0.001) and overweight/obesity (*a*_4_ = −0.098; comparison to normal weight, *p* = 0.030) show stronger effects than normal weight (*a*_4_ = −0.014) for incongruence of motor competence and physical self-concept. That means, higher scores in both predictors as well as greater accuracy between motor competence and physical self-concept predict more future physical activity, especially for the unhealthy weight groups underweight and overweight/obesity. This trend followed the rule also if overweight and obesity were analyzed separately (for detailed analyses^36^). The effect was stronger for obese children compared to overweight ones. However, due to the small sample of obese children (*n* = 36), this conclusion is a preliminary one as it is based on low power.

## Discussion

Both proficiency in motor competence and a favourable physical self-concept are positively associated with physical activity and consequently with a lower risk of obesity in childhood^5,13,27^. Previous studies suggest moderate effects when—mostly cross-sectionally—investigating the two main effects of motor competence^15,16,37^ and physical self-concept^24^ on physical activity in childhood. Going beyond previous studies, we were particularly interested in both the main effects and, additionally, the specific interplay of the two predictors. Consequently, we aimed at investigating the effect of motor competence, the effect of physical self-concept, and their simultaneous effect of an (in-) accurate physical self-concept on future physical activity behavior in middle childhood by modeling the accuracy of the physical self-concept and motor competence levels as additional predictor for physical activity.

Importantly, all variables showed substantial stability from 3^rd^ to 4^th^ grade, suggesting a reliable assessment on the one hand, but nevertheless giving room for a successful prospective prediction of physical activity on the other. Indeed, the results showed main effects for both predictors. Higher scores on physical self-concept as well as higher levels of motor competence were positively associated with physical activity one year later for all children (linear additive effect). Thus, our results are in line with the well-known conceptual model of Stodden *et al*.^13,27^.

Moreover, we found evidence for a so-called agreement (or fit/congruence) effect between actual motor competence and the associated physical self-concept. This means, compared to higher values on each scale observed in an isolated fashion, the more accurate motor self-perceptions are, the higher are physical activity levels one year later. Over- as well as underestimating one’s own motor competence both lead to lower future physical activity levels for these children. In essence, these results suggest putting actual motor competence, physical self-concept, but especially the fit pattern between both (congruency) in the focus of promoting physical activity behaviors of children.

Detailed analyses revealed that the agreement effect is moderated by weight status in a U-form (to a second degree). The effects reported were stronger for unhealthy weight groups such as underweight and overweight/obesity compared to normal weight children. In these groups, the results indicate that exclusively promoting self-perceptions, based on the fact that higher perceived scores are associated with higher physical activity levels, which leads to overestimation of one’s own motor competence, cannot be recommended. Similarly, exclusively promoting their motor competence can also not be recommended because not only the main effect but also the accuracy effect affect future physical activity. For the majority of normal weight children, however, both factors rather add up in terms of more activity.

In other studies, the overestimation of one’s own competencies is sometimes regarded as being beneficial for psychological health or physical activity^31^. In most studies conclusions are drawn based on clustering groups and difference scores between self-perception and actual competence. However, difference scores that identify an over- or underestimator do not provide information about actual levels of motor competence or physical self-concept and can lead to further measurement errors^38^. To illustrate this issue for two overestimators, who were classified based on a simple difference score: Person one could have low motor competence and an average physical self-concept, whereas person two could have average motor competence and a high physical self-concept. Controlling for the level of both predictors, the polynomial regression with response surface analysis suggests two main effects and an additional agreement effect, indicating that higher levels of both predictors are beneficial for future physical activity, but, under- or overestimating one’s own motor competence both lead to lowered physical activity levels.

In general, some children might accurately estimate their motor competence level. However, over- as well as underestimation do not just randomly occur. Children integrate various experiences such as mastery, internal, and external feedback into their physical self-concept levels. Consequently, a distribution of perception errors across children occurs. In early childhood, children are normally overestimators, because they rely on the delusion of grandeur and perceive their own competence as higher than it actually is based on external, mostly parental, feedback. While growing up, obese, overweighted, and underweighted children often receive stigmated feedback in their daily lives^39^. For instance, overweight and obese children get intimidated and bullied around in sport settings^40,41^, differ in body (self-)perceptions compared to normal weight children^42^, and are regarded as being unathletic. Underweight children are, in contrast, generally regarded as being good at sports. Thus, they experience throwbacks in sports, because they are not as good as they (are) thought.

The received feedback affects children’s general as well as motor self-perceptions in one or the other way. Importantly, children are usually unaware that they are over- or underestimating. While children prefer engaging in areas where they perceive themselves as being competent—for instance in sports and exercise in case of high physical self-concepts—competence theory also states that missing success or a lack of accomplishments leads to avoidance behavior in that specific area. This demonstrates the importance of the underlying mechanisms of mastery attempts in childhood, where success or failure determine in which domain, activity, game, or sports children will engage. Not knowing one’s own competence and according inaccurate self-views, as well as experiencing failure and according deficits can destroy the facade children in these weight groups may have tried to create, revealing that their perception is in fact a fuzzy version of their actual self. Self-enhancement, for instance, tends to occur more often in specific domains that are most important to persons, and less often in the more peripheral, less important domains^43^. This is why one aim of working with children and the improvement of their actual and perceived motor competence should be enjoyment and self-knowledge to centralize these topics in their daily lives already in early childhood.

The main strength of this study is the longitudinal investigation of important associations, using a complex and adequate statistical analysis, which controls and especially models accuracy effects accounting for level information in both predictors. Within the analyses, all variance is reflected in the results compared to cluster analytical procedures, where persons are grouped into categories. The large sample size of this study led to high power. Nevertheless, this study is only a first step and future research should replicate, develop, and apply the presented approach in parallel datasets as well as in other age groups. A limitation is that physical activity was assessed using a self-reported questionnaire, which needs to be extended to objective measurements (e.g., accelerometers)^44^. Furthermore, motor assessments should be extended to include locomotor skills, process- as well as product-oriented motor assessments in the future. Future research should also investigate the interplay of physical fitness parameters and related self-perceptions.

In conclusion, the current findings provide an initial step towards a differentiated investigation of the physical self-concept and actual motor performance and their interplay on future physical activity in children. Introducing the polynomial regression with Response Surface Analysis as a relatively new and powerful tool to investigate agreement/congruency/accuracy hypotheses to the motor development and physical activity research fields, the results underline two main conclusions: (1) they underline the relevance of main effects of physical self-concept and actual motor performance on physical activity, (2) they underline, above and beyond the main effects and baseline physical activity^36^, a meaningful effect of the accuracy of self-views, especially in unhealthy weight groups. This study highlights the importance of comprehensive sport programs for children to increase motor competence and physical self-concept levels on the one hand and to achieve accurate self-knowledge about their motor competence levels on the other hand.

## Methods

### Participants and procedure

In total, 718 students from the 3^rd^ and, one year later, in the 4^th^ grade (age 3^rd^ grade: *M* = 9.0 years, *SD* = 0.72; 48.2% female) participated in the study both years in all relevant assessments. Data was collected within the project ‘Healthy Children in Sound Communities’ (i.e., HCSC; for the whole study design, see^45^). HCSC took place in Germany and the Netherlands from 2008 to 2014 and is a longitudinal study that accompanies the psycho-social and motor development of primary school aged students. Participants were located in rural and urban areas in the west of Germany and in the east of Netherlands. Participating communities and schools were selected based on representative migration background (12% in this sample) and diverse socio-economical status. Thus, the present sample can be regarded as representative for western European children.

The standardized data collection was conducted by trained teams of research assistants in a classroom setting (normal school classroom as well as sport gyms) within the schools themselves. The study procedure was approved by the ethics committee of the European Union. All methods were performed in accordance with the relevant guidelines and regulations. For each participant, a written informed consent was obtained from a parent or legal guardian and each participant could stop participating at any time.

### Materials

The body mass index (BMI) of each 3^rd^ grade child was calculated based on height and weight. Based on age- and sex-specific information of the WHO guidelines^46^, the raw BMI values of the children were then assigned to one of the four weight groups 3^rd^ grade: underweight (*n* = 55), normal weight (*n* = 494), overweight (*n* = 130), and obese (*n* = 36). Three participants did not provide weight or height information.

Motor competence was assessed via three validated object control (ball) skills from the General Sportmotoric Test for Children [Allgemeiner Sportmotorischer Test für Kinder]^47^ and the Selectiontest for Remedial Physical Education [Auswahltest für den Sportförderunterricht]^48^. This test battery was specifically composed for the project to assess product-oriented ball skills^49^. For each participant, a composite score was calculated, including the three items: throwing a ball at a target (target throwing), bouncing a ball (bouncing), throwing a ball through the legs against a wall and catching it (throw and catch), while controlling for sex and age as covariates. Target throwing consisted of five trials of throwing a ball at a target zone. Participants received points for accuracy for each trial. Bouncing was assessed via the number of bounces of a gymnastic ball within 30 seconds. The performance on the throw and catch item was assessed using a six-points scale for the qualitative execution. Considering the briefness of the scale, reliability for motor competence in this study was good (Cronbach’s α = 0.65).

Physical self-concept was assessed via the sportiness self-perception scale of the physical self-concept questionnaire, which has been shown to be reliable and valid for this age group^50^. Accordingly, physical self-concept is operationalized as a self-perception of sport competence via three items (i.e., “I am good at sports”, “I am a good athlete”, “I am very athletic”) on a four-points Likert scale (1 = *not true* to 4 = *very true*). A composite score was calculated out of the three items. Considering the briefness of the scale, the reliability in this sample was good (Cronbach’s α = 0.72).

Physical activity was assessed via a self-report questionnaire covering four components: commute to school, sports participation, leisure time physical activity with friends, and leasure time physical activity alone. Each question (e.g., “How often do you play outside?”; For all questions, see supplementary material) was assessed using a four-points Likert scale (1 = *never* to 4 = *always*). Physical activity was operationalized via a coefficient covering all items according to the protocol^45^. Again, considering the nature of the scale, the reliability in this study was good (Cronbach’s α = 0.72).

### Data analysis

Statistical analyses were conducted using *R*^51^ (for open code and open data^36^) and primarily the *RSA* package^52^.

First, the stabilities of physical activity, motor competence, and physical self-concept are examined using bivariate pearson correlation coefficients. Second, both predictor variables, motor competence and physical self-concept in 3^rd^ grade, are z-transformed. The outcome variable is the physical activity composite score in 4^th^ grade. Third, to analyze the effects of motor competence and physical self-concept on physical activity (i.e., two main effects and the interplay of both), a second degree polynomial regression with response surface analysis (RSA)^52^ was applied. The RSA analyses the effects of fit patterns of two predictor variables on one outcome variable using a path modeling approach. Fit patterns can hereby be defined as the optimal match (agreement/accuracy/fit) between the levels of two variables. It is controlled for outliers according to the criteria introducted by Bollen and Jackman^53^. The polynomial regression of the second degree is estimated using the following equation (1):

1 $$\begin{array}{c}\,physical\,activity\, \sim \,{b}_{1}\ast physical\,selfconcept+{b}_{2}\ast motor\,competence\\ \,\,\,\,\,\,\,\,+\,{b}_{3}\ast physical\,selfconcep{t}^{2}+{b}_{4}\ast motor\,competence\ast physical\,selfconcept\\ \,\,\,\,\,\,\,\,+\,{b}_{5}\ast motor\,competenc{e}^{2}\end{array}$$

Bootstrapped confidence intervals are computed for all regression coefficients. Regression coefficients are *b*_1_ to *b*_5_. Additionally, regression coefficients *a*_1_ to *a*_4_ are estimated in the model. Comparable with linear regressions, the linear main effects are *b*_1_ (physical self-concept) and *b*_2_ (motor competence). The curvilinear main effects are *b*_3_ (physical self-concept) and b_5_ (motor competence), and the interaction effect is *b*_4_ (motor competence * physical self-concept). The squared and interaction terms of the predictor variables are modeled using the maximum likelihood estimator (ML) and robust standard errors, which are robust against violations of the assumption of normality. Further modeled effects, *a*_1_ to *a*_4_, provide detailed information on specific effects of the interplay of the predictor variables: (i) the linear effect on the line of congruence, *a*_1_ (LOC), which gives insight into the linear interplay of the two predictors, (ii) the curvature effect on the LOC, *a*_2_, which reflects a quadratic interplay of the two predictors, (iii) the coefficient *a*_3_, which gives insight into whether or not a ridge is being shifted away from the LOC, and (iv) the coefficient *a*_4_ (LOIC), which reflects the general effect of incongruence above the line of incongruence. An optimal fit pattern can be analysed by determining specific conditions between the predictor variables. For instance, one important condition for an incongruence or congruence effect is a significant *a*_4_ coeficient (for a discussion^54^).

Fourth, within the modeling process, several models with specific patterns of the regression coefficients are estimated and compared. To avoid the selection of over- and underfitting models, the following procedure is used. Relative model fit is examined using Akaike’s Information Criteria (for an overview see^55^), because nested and non-nested models are compared. The Akaike information criterion (AICc) index is sensible to over- and underfitting, because it adjusts the predictive accuracy of a model relative to its complexity (parsimony). Models within a range of three AIC-points are considered to be essentially equal. Models can be compared using χ^2^ statistics, using adjusted *R*^2^s, comparative fit indices (if CFI > 0.9), and degrees of freedom (parismony of the model). All of these parameters are taken into account for model selection and can be interpreted as standardized regression coefficient. If two or more models fit the data equally well, the more parsimonious model will be preferred.

Fifth, a quasi DIF approach is used to examine effect origins of the resulting model, comparing the weight groups underweight, normal weight, overweight/obesity. These stratifications of body mass index are used to operationalize weight status as objectively as possible based on meaningful WHO norms that account for gender and age on the basis of a quarter year. Differences are calculated using z-tests^56^. Post-hoc power of the results was simulated via a bootstrapping procedure after the modeling process for each regression coefficient.

## Electronic supplementary material

Supplement

Supplementary Dataset 1
