# Supplementary material for: Understanding physical (in-) activity, overweight, and obesity in childhood: Effects of congruence between physical self-concept and motor competence
Source: Sci Rep. 2018 Apr 12;8:5908. doi: 10.1038/s41598-018-24139-y (PMC5897370; doi:10.1038/s41598-018-24139-y)
Supplement: Supplementary file 1 — Supplement [file 41598_2018_24139_MOESM1_ESM.docx]

**Supplement**

**Understanding physical (in-)activity, overweight, and obesity in childhood: Effects of congruence between physical self-concept and motor competence**

Utesch, T.^1*^, Dreiskämper D.^1,2^, Naul, R.^1,2^, & Geukes, K.^1^

^1^ University of Münster, Germany

^2^ Willibald Gebhardt Institute, Germany

*Contact information of the corresponding author:

Dr. Till Utesch

Horstmarer Landweg 62b

48149 Münster

Germany

Tel. +49-251-83-31805

Mail: till.utesch@uni-muenster.de

**Supplement: Physical Activity Questionnaire**

**1) Way to school.**

*Please mark how you get to school and back.*

|  | *Never* | *Rarely* | *Often* | *Always* |
| --- | --- | --- | --- | --- |
| I take the bus. | *O* | *O* | *O* | *O* |
| I am taken by car. | *O* | *O* | *O* | *O* |
| I take the bicycle. | *O* | *O* | *O* | *O* |
| I walk. | *O* | *O* | *O* | *O* |

**2) Leisure time with friends.**

When you meet up with your friends, what do you do?

*Please mark what you do and how often.*

|  | *Never* | *Rarely* | *Often* | *Always* |
| --- | --- | --- | --- | --- |
| Playing outside | *O* | *O* | *O* | *O* |
| Riding the bicycle | *O* | *O* | *O* | *O* |
| Skating (inline skates or skateboard) | *O* | *O* | *O* | *O* |

**3) Leisure time alone.**

Sometimes none of your friends got time.

*Please mark what you do when you are alone.*

|  | *Never* | *Rarely* | *Often* | *Always* |
| --- | --- | --- | --- | --- |
| Playing outside | *O* | *O* | *O* | *O* |
| Riding the bicycle | *O* | *O* | *O* | *O* |
| Skating (inline skates or skateboard) | *O* | *O* | *O* | *O* |

**4) Types of sports.**

There are different types of sport.

*Please mark how often you participate in the following sports, every time at least 20 minutes.*

|  | *Never* | *Rarely* | *Often* | *Always* |
| --- | --- | --- | --- | --- |
| Swimming | *O* | *O* | *O* | *O* |
| Riding the bicycle | *O* | *O* | *O* | *O* |
| Running | *O* | *O* | *O* | *O* |
| Ball sports  (Football, volleyball, handball) | *O* | *O* | *O* | *O* |
| Badminton, Tennis | *O* | *O* | *O* | *O* |
| Skating (inline skates or skateboard) | *O* | *O* | *O* | *O* |
| Table tennis | *O* | *O* | *O* | *O* |
| Horseback riding | *O* | *O* | *O* | *O* |
| Dancing | *O* | *O* | *O* | *O* |

**Supplement: CODE**

####################################

## Response Surface Analysis: Interplay of Perceived and Actual Motor Competence on Physical Activity

## by Dr. Till Utesch

## Data from Project Healthy Children in Sound Communities (Naul et al., 2012)

## Children age 9-10-years old

####################################

## Operationalization

## Physical self-concept dimension: Self-Concept Sportiness based on Dreiskämper et al. 2015 (PSK)

## Motor Competence: Object Control (3 items) based on the importance of object control for future physical activity (cf. Barnett et al., 2008): Target Throwing, Ball Bouncing, Ball-Legs-Wall

## Physical Activity Questionnaire based on HCSC study

###############

##

## install and load packages

##

###############

#install.packages("RSA")

#install.packages("qgraph")

#install.packages("haven")

#install.packages("rgl")

#install.packages("psych")

#install.packages("dplyr")

#install.packages("GPArotation")

#install.packages("QuantPsyc")

library(rgl)

library(haven)

library(qgraph)

library(RSA)

library(psych)

library(dplyr)

library(GPArotation)

library(QuantPsyc)

###############

##

## Prepare data

##

###############

# set path

setwd("/Users/..../R/RSA")

# load data from spss file

data<-read_spss("responsesurfaceanalysis_data.sav")

names(data)

# create subdata sets for analysis ####

pa <- data %>% dplyr::select(45:63) # subset for the reliability analysis for physical activity

mc <- data %>% dplyr::select(64:72) # subset for the reliability analysis for motor competence

psk <- data %>% dplyr::select(psk1_grade3, psk2_grade3, psk3_grade3) # subset for the reliability analysis for physical self-concept

underweight <- data %>% filter(BMI_Cole_grade3 == 1) # subset for the RSA with underweight children

normalweight <- data %>% filter(BMI_Cole_grade3 == 2) # subset for the RSA with normal weight children

overweight <- data %>% filter(BMI_Cole_grade3 == 3) # subset for the RSA with overweight children

obesity <- data %>% filter(BMI_Cole_grade3 == 4) # subset for the RSA with obese children

overobese <- data %>% filter(BMI_Cole_grade3 > 2) # subset for the RSA with overweight and obese children

## reliability analysis ####

alpha(pa) # alpha = .72 (CI95: .69; .75) for physical activity

alpha(mc) # alpha = .65 (CI95: .64; .71) for motor competence

alpha(psk) # alpha = .72 (CI95: .69; .76) for physical self-concept

#### Stability analysis ####

names(data)

stability <- data %>% dplyr::select(selfconcept_grade3, selfconcept_grade4, objectcontrol_grade3, objectcontrol_grade4, physicalactivity_grade3, physicalactivity_grade4)

### STABILITY OF THE CONSTRUCTS

pairs.panels(stability)

r <- corr.test(stability)

r

# object control, r = .37, p < .001

# self-concept, r = .54, p < .001

# physical activity, r = .55, p < .001

###############

##

## Run RSA Analysis

##

###############

# RSA analysis for the whole sample ####

# compute model

RSA_fullsample<-RSA(physicalactivity_grade4~selfconcept_grade3*objectcontrol_grade3, data= data, na.rm = T, center=T, scale=TRUE)

## compare models and choose best models

aictab(RSA_fullsample) # best models are model RR, SRRR, full

# after comparision, RR model was chosen

# summary of results RR model

summary(RSA_fullsample, model="RR")

# plot surface of RR model

plot(RSA_fullsample, model="RR", xlab="Self-Perception", ylab="Motor Competence", zlab="Physical Activity", points=list(show=F))

## for supplementary analyses:

summary(RSA_fullsample, model="SRRR")

plot(RSA_fullsample, model="SRRR", xlab="Self-Perception", ylab="Object Control", zlab="Physical Activity", main = "SRRR Model", points=list(show=F))

summary(RSA_fullsample, model="full")

plot(RSA_fullsample, model="full", xlab="Self-Perception", ylab="Object Control", zlab="Physical Activity", main = "Full Model", points=list(show=F))

### Step 4: Analysis of weight influence ####

## RSA model for underweight children

RSA_underweight<-RSA(physicalactivity_grade4~selfconcept_grade3*objectcontrol_grade3, data= underweight, na.rm = T, center=T, scale=TRUE)

summary(RSA_underweight, model="RR")

plot(RSA_underweight, model="RR", xlab="Self-Perception", ylab="Object Control", zlab="Physical Activity", points=list(show=F), cex = 2)

# RSA model for normal weight children

RSA_normal<-RSA(physicalactivity_grade4~selfconcept_grade3*objectcontrol_grade3, data= normal, na.rm = T, center=T, scale=TRUE)

summary(RSA_normal, model="RR")

plot(RSA_normal, model="RR", xlab="Self-Perception", ylab="Object Control", zlab="Physical Activity", points=list(show=F), cex = 2)

# RSA model for overweight and obese children

RSA_overobese<-RSA(physicalactivity_grade4~selfconcept_grade3*objectcontrol_grade3, data= overobese, na.rm = T, center=T, scale=TRUE)

summary(RSA_overobese, model="RR")

plot(RSA_overobese, model="RR", xlab="Self-Perception", ylab="Object Control", zlab="Physical Activity", points=list(show=F), cex = 2)

### checking whether the trend continues for obese children (do not overinterpret, because of small sample size)

# RSA model for overweight children

RSA_overweight<-RSA(physicalactivity_grade4~selfconcept_grade3*objectcontrol_grade3, data= overweight, na.rm = T, center=T, scale=TRUE)

summary(RSA_overweight, model="RR")

plot(RSA_overweight, model="RR", xlab="Self-Perception", ylab="Object Control", zlab="Physical Activity", main = "OVERWEIGHT", points=list(show=F))

## RSA model for obese children

RSA_obesity<-RSA(physicalactivity_grade4~selfconcept_grade3*objectcontrol_grade3, data= obesity, na.rm = T, center=T, scale=TRUE)

summary(RSA_obesity, model="RR")

plot(RSA_obesity, model="RR", xlab="Self-Perception", ylab="Object Control", zlab="Physical Activity", main = "OBESSITY", points=list(show=F, value="predicted"))

### trend continous althought power will be low due to sample size.

###

#

# Significance test between regression weights of different weight groups

# based on z-tests

# Paternoster, R., Brame, R., Mazerolle, P., & Piquero, A. R. (1998). Using the Correct Statistical

# Test for the Equality of Regression Coefficients. Criminology, 36(4), 859–866.

#

###

### linear effects significance test

bun <- 0.083

bunse <- 0.027

bno <- 0.035

bnose <- 0.012

bovbe <- 0.076

bovbese <- 0.018

testb1 <- (bno-bun)/(sqrt(bnose^2+bunse^2))

print(testb1)

testb2 <- (bno-bovbe)/(sqrt(bnose^2+bovbese^2))

print(testb2)

### LOC effect significance test

locun <- 0.166

locunse <- 0.054

locno <- 0.070

locnose <- 0.023

locovbe <- 0.152

locovbese <- 0.036

testloc1 <- (locno-locun)/(sqrt(locnose^2+locunse^2))

print(testloc1)

testloc2 <- (locno-locovbe)/(sqrt(locnose^2+locovbese^2))

print(testloc2)

# LOIC effect significance test

ovbe <- -0.098

ovbese <- 0.039

no <- -0.014

nose <- 0.022

un <- -0.259

unse <- 0.063

testloic1 <- (no-un)/(sqrt(nose^2+unse^2))

print(testloic1)

testloic2 <- (no-ovbe)/(sqrt(nose^2+ovbese^2))

print(testloic2)

########################################

############################################

#####

#####

##### Post-hoc Power Calculation for RSA

#####

#####

#############################################

########################################

#install.packages("mvtnorm")

library(mvtnorm)

## Simulate the results

## what is the r-squared of the regression model?

r2 <- 0.052

## define the means and the correlations between the variables

## (note: variables were z-standardized prior to the analyses)

correl <- cor(fullsample$objectcontrol_grade3, fullsample$selfconcept_grade3, method = c("pearson"))

correl

obs_mean <- c(0,0)

obs_corr <- matrix(c(1,0.2171445,0.2171445,1),2,2)

N <- 718

## generate the samples and check whether the coefficients are significant or not

out <- data.frame()

reps <- 1000 # numbers of replications for the simulation

for (i in 1:reps) {

## generate random sample

p <- rmvnorm(N,mean=obs_mean,sigma=obs_corr)

zp <- 2.385+0.047*p[,1]+0.047*p[,2]+(-0.014)*p[,1]^2+(-0.014)*p[,2]^2+0.027*p[,1]*p[,2]

z <- zp + rnorm(N,0,sqrt(((1-r2)*var(zp))/r2))

randomsample <- data.frame(z=z,x=p[,1],y=p[,2],x2=p[,1]*p[,1],y2=p[,2]*p[,2],xy=p[,1]*p[,2])

## estimate regression model

m <- lm(z~x+y+x2+x*y+y2,data=randomsample)

pss <- summary(m)[4]$coefficients[,4]

zw <- data.frame(b0=coef(m)[1],b1=coef(m)[2],b2=coef(m)[3],

b3=coef(m)[4],b4=coef(m)[5],b5=coef(m)[6],

p.b0=pss[1],p.b1=pss[2],p.b2=pss[3],

p.b3=pss[4],p.b4=pss[5],p.b5=pss[6])

out <- rbind(out,zw)

}

## Check means of estimated coefficients (the mean should be similar to the

## original values)

colMeans(out[,1:6])

# Post-hoc power values

(sum(out$p.b1 <= 0.05)/reps)*100 # power b1

(sum(out$p.b2 <= 0.05)/reps)*100 # power b2

(sum(out$p.b3 <= 0.05)/reps)*100 # power b3

(sum(out$p.b4 <= 0.05)/reps)*100 # power b4

(sum(out$p.b5 <= 0.05)/reps)*100 # power b5

### Supplementary: LINEAR REGRESSIONS TO CONTROL FOR BASELINE PHYSICAL ACTIVITY ####

# linear regression with two main effects, but without physical activity baseline in the model

linreg_2maineffects <- lm(physicalactivity_grade4 ~ objectcontrol_grade3 + selfconcept_grade3, data=data)

summary(linreg_2maineffects)

#standardized coefficients

stdcoefficients <- lm.beta(linreg_2maineffects)

stdcoefficients[1] # object control

stdcoefficients[2] # self-concept

# both main effects are significant on a one-sided test with

# object control, b = .085, p = .02

# self-concept, b = .18, p < .001

# linear regression with physical activity baseline in the model:

linreg_3maineffects <- lm(physicalactivity_grade4 ~ objectcontrol_grade3 + selfconcept_grade3 + physicalactivity_grade3, data=data)

summary(linreg_3maineffects)

stdcoefficients1 <- lm.beta(linreg_3maineffects)

stdcoefficients1[1] # object control

stdcoefficients1[2] # self-concept

stdcoefficients1[3] # physical activity

# all three main effects are significant on a one-sided test. However, multicovariance reduces predicitve power of self-concept and marginally from object control.

# object control, b = .061, p = .0265

# self-concept, b = .054, p = .047

# physical activity, b = .54, p < .001
